# Successful Management of Exposure Keratitis Using a Simple Eye Band (SEB) in ICU Patients Unwilling to Undergo Surgical Tarsorrhaphy

**DOI:** 10.7759/cureus.100316

**Published:** 2025-12-29

**Authors:** R Balamurugan, Sharada V Kutty, Timitrov P, Shubhangi SN Prasad, Kiratmeet Singh

**Affiliations:** 1 Ophthalmology, All India Institute of Medical Sciences, Madurai, Madurai, IND; 2 Internal Medicine, All India Institute of Medical Sciences, Mangalagiri, Mangalagiri, IND; 3 Nephrology, All India Institute of Medical Sciences, Mangalagiri, Mangalagiri, IND; 4 Ophthalmology, All India Institute of Medical Sciences, Mangalagiri, Mangalagiri, IND

**Keywords:** disposable device, exposure keratitis, simple eye band, temporary tarsorrhaphy, unconscious patient

## Abstract

Exposure keratitis is a common yet potentially sight-threatening ocular complication that occurs in unconscious patients in intensive care unit (ICU) and emergency settings. Various adhesive and surgical techniques have been used for its management. Adhesives may not be effective in cases of repeated eyelid opening, and surgical tarsorrhaphy may not be suitable for many patients who are unfit for the procedure. Additionally, both adhesives and sutural tarsorrhaphy can limit repeated ocular examinations and the frequent instillation of medications. To address these challenges, we devised a simple homemade cotton gauze band named the simple eye band (SEB) and successfully managed two cases of exposure keratitis in patients who were unwilling to undergo surgical tarsorrhaphy. The SEB has the advantage of being easily opened and closed, similar to opening a book, allowing repeated ocular examinations and the instillation of eye drops without the need for adhesives or invasive procedures.

## Introduction

Exposure keratitis (EK) is a sight-threatening ocular complication frequently encountered in unconscious, sedated, or critically ill patients in emergency and intensive care unit (ICU) settings. It occurs due to lagophthalmos and incomplete eyelid closure, which result in improper tear-film spread over the cornea and accelerated evaporation from the exposed ocular surface [1]. The early manifestations typically include superficial punctate erosions accompanied by ocular surface dryness; however, in the absence of timely preventive or therapeutic interventions, these lesions may rapidly progress to persistent epithelial defects, secondary microbial infections, stromal thinning, corneal melt, and, in severe cases, irreversible vision loss [1,2]. Temporarily closing the eyelids in such patients is crucial for preventing and treating exposure keratopathy, and clinicians generally rely on adhesive techniques such as adhesive tape closure [1], the application of eye patches [1], tape splint tarsorrhaphy [3,4], cyanoacrylate [5], and moist chamber [6] or on sutural methods like temporary tarsorrhaphy [1] and drawstring temporary tarsorrhaphy [7]. Although these methods are used commonly, they are accompanied by notable drawbacks: adhesive tapes may fail to achieve complete eyelid closure, especially in patients requiring frequent instillation of topical medications or repeated ocular examinations, and may cause skin irritation. Similarly, tarsorrhaphy, while effective in achieving lid closure, is invasive, may deform the eyelids, necessitates wound care and dressing maintenance, requires repetition in longstanding or persistently unconscious patients, and significantly limits the ability of clinicians to perform essential ophthalmic assessments such as evaluation of pupillary reactions, assessment for relative afferent pupillary defect (RAPD), and instillation of therapeutic eye drops. Sometimes, patients or their guardians may not be willing to provide consent for invasive tarsorrhaphy, or the patients may not be fit for the surgical tarsorrhaphy. To address these limitations, we developed a simple, homemade, non-invasive, non-adhesive, cotton gauze-made eye band named the “simple eye band (SEB),” designed to ensure effective eyelid closure, especially in unconscious ICU patients, without the need for adhesives or invasive procedures while allowing repeated easy access to the eyes for examination and instillation of drops, thereby helping in the prevention and treatment of EK.

## Case presentation

**Figure 1:**
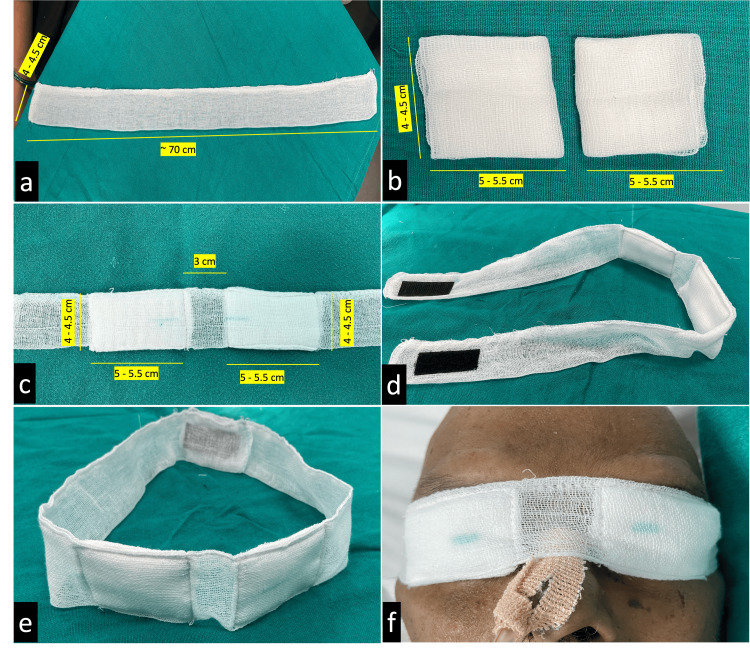
(a) A double-layered gauze strap measuring approximately 70 cm in length and 4–4.5 cm in breadth. (b) Two four-layered eye pads measuring 5–5.5 cm and 4–4.5 cm. (c) The two eye pads stitched to the strap, separated by 3 cm. (d) Side view of the SEB with the Velcro attached. (e) Anterior view of the SEB. (f) Application of the SEB over the closed eyelid SEB: simple eye band

The SEB is a disposable, easy-to-fabricate eye band consisting primarily of two components: (i) a single strap measuring approximately 70 cm (27.5″) in length and 4-4.5 cm (1.6-1.8″) in width (Figure 1a) and (ii) two four-layered eye pads, each measuring approximately 5-5.5 cm × 4-4.5 cm (2.0-2.2″ × 1.6-1.8″) (Figure 1b), stitched onto the strap at a distance of 3 cm (1.2″) from each other (Figure 1c) to align naturally with the eyelids when placed over the eyes. Velcro strips may be attached to its ends (Figures 1d, 1e) to facilitate easy fastening and unfastening. All measurements are approximate and can be customized based on the patient’s facial dimensions. The SEB can be sterilized by autoclaving and is intended for single-day use. Application involves placing the two padded sections over the patient’s closed eyelids and securing the strap around the head (Figure 1f); excess strap length can be adjusted by tying a simple knot (Video 1). During eye examinations or instillation of medications, the Velcro end can be opened like a flap, providing immediate access to the ocular surface, and resealed afterward to maintain eyelid closure. There should not be excessive tightening of the band; to ensure this, a single finger should be able to be inserted beneath the band at the side of the head. After securing the strap, intraocular pressure (IOP) was grossly checked using digital palpation IOP assessment. In this report, we describe the successful management of two patients with EK using our SEB, whose guardians declined sutural tarsorrhaphy in the ICU.

**Video 1:** Application of the simple eye band (SEB) in an unconscious patient with exposure keratitis The authors obtained signed written consent for the publication of the video from the patient’s daughter, as the patient has passed away

Case 1

**Figure 2:**
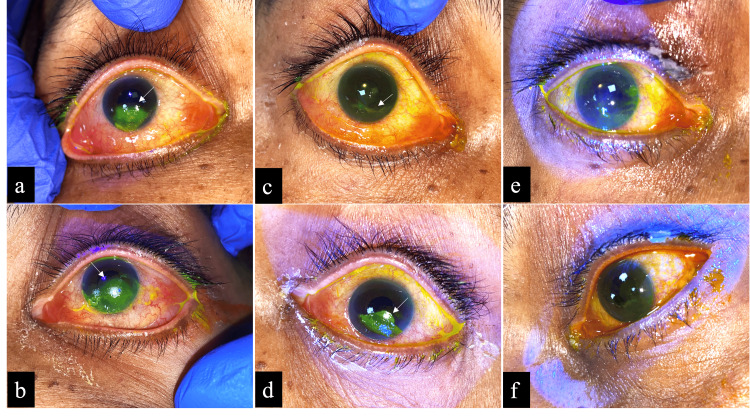
(a, b) Exposure keratitis of the right and left eyes in Case 1, stained with fluorescein dye, measuring approximately 4 × 7 mm in the right eye and 6 × 7 mm in the left eye. (c, d) Reduction in the size of exposure keratitis in both eyes, leaving only a streak-like lesion in the right eye and a 2 × 4 mm lesion in the left eye on Day 1, after the application of SEB. (e, f) Complete disappearance of exposure keratitis in the right eye, with a mild residual streak in the left eye by Day 6 SEB: simple eye band

A 61-year-old male patient with traumatic brain injury, paraparesis, and poor sensorium was mechanically ventilated and developed EK measuring approximately 4 × 7 mm in the right eye (Figure 2a) and 6 × 7 mm in the left eye (Figure 2b) on fluorescein staining, despite using adhesive patches for many days. Upon applying the SEB, complete closure of the eyelids was confirmed from an inferior view (Video 1). Remarkably, by Day 1, the epithelial defect had reduced to a streak-like lesion in the right eye (Figure 2c) and a 2 × 4 mm lesion in the left eye (Figure 2d), and by Day 6, the right eye exhibited complete healing (Figure 2e), while the left eye showed only a mild residual streak (Figure 2f). There was no follow-up after this, as the patient passed away due to a systemic illness.

Case 2

**Figure 3:**
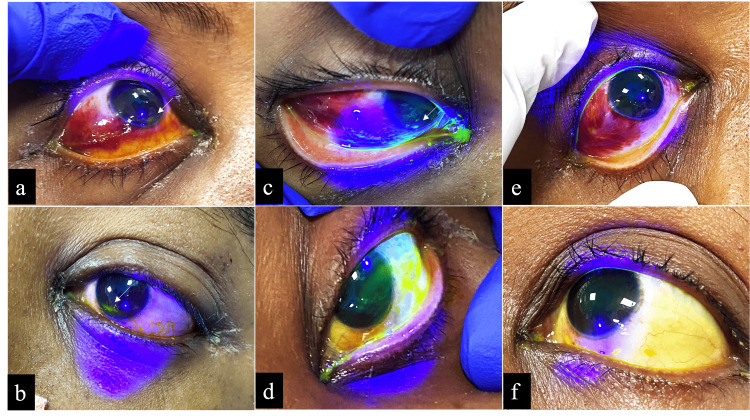
(a, b) Exposure keratitis of the right and left eyes, stained with fluorescein dye, measuring approximately 2 x 0.5 mm in both eyes. (c, d) Reduction in the size of exposure keratitis to a 0.5 mm streak in the right eye and complete disappearance in the left eye on Day 1. (e, f) Complete disappearance of exposure keratitis in both eyes by Day 3

A 26-year-old female patient admitted with scrub typhus-related multiorgan dysfunction, intubated and mechanically ventilated with intermittent sedation breaks, also developed bilateral EK measuring approximately 2 × 0.5 mm (Figures 3a, 3b), despite using adhesive patches for many days. After application of the SEB, the defects decreased to a 0.5 mm streak in the right eye (Figure 3c) with complete resolution in the left eye (Figure 3d) by Day 1, and complete healing in both eyes (Figures 3e, 3f) was observed by Day 3.

Both cases were given prophylactic antibiotics and lubricating eye drops six times a day.

## Discussion

Timely intervention for EK is crucial to prevent its complications, and in some situations, patients or their guardians may be unwilling to consent to the procedure or the patient may be unfit for surgical tarsorrhaphy [1,2]. The SEB offers an alternative option to surgical tarsorrhaphy, as it is non-invasive and allows frequent examination of the eyes and instillation of topical medications. In our two cases, we appreciated the clinical usefulness of the SEB as a non-invasive, non-adhesive, easily applicable, and customizable band that provides effective eyelid closure for both prevention and treatment while allowing repeated ocular evaluations, medication administration, and monitoring of lesions. The SEB can be inexpensively fabricated within hospital settings and customized as needed, making it a practical and scalable solution for ICU conditions. It may serve as an alternative to invasive tarsorrhaphy in patients who are not fit for surgery, those on anticoagulants, or those unwilling to undergo sutural tarsorrhaphy. The application of the SEB can be easily taught to nursing staff, enabling them to apply it independently without the need for an ophthalmologist. We did not encounter any adverse events with the SEB. However, excessive pressure on the globe is a potential concern; therefore, the strap should be applied with ideal tightness. This can be ensured by allowing insertion of one index finger beneath the band at the side of the head to avoid excessive tightening. Additionally, IOP can be grossly assessed using digital palpation IOP assessment at the end of SEB application.

## Conclusions

EK is a preventable ocular surface disease that commonly affects unconscious or critically ill patients in ICU settings, and early preventive strategies are essential to avoid secondary complications. The SEB, being a homemade, non-invasive, non-adhesive, and disposable cotton gauze band that can be applied without an ophthalmologist, offers a promising method for both the prevention and management of EK. Our early cases demonstrate promising clinical outcomes; however, larger case series and randomized controlled trials are required to establish its efficacy in comparison with other treatment modalities. This technique may also be considered in pediatric patients as a less invasive alternative to tarsorrhaphy, particularly in situations where surgical intervention is contraindicated or not feasible.
